# The link between rheumatic disorders and inborn errors of immunity

**DOI:** 10.1016/j.ebiom.2023.104501

**Published:** 2023-03-02

**Authors:** Georgios Sogkas, Torsten Witte

**Affiliations:** a Department of Rheumatology and Immunology, Hannover Medical University, Hannover, Germany; b Hannover Medical School, Cluster of Excellence RESIST (EXC 2155), Hannover, Germany

**Keywords:** Inborn errors of immunity, Autoimmunity, Autoinflammation, Rheumatoid arthritis, Systemic lupus erythematosus, Spondyloarthritis

## Abstract

Inborn errors of immunity (IEIs) are immunological disorders characterized by variable susceptibility to infections, immune dysregulation and/or malignancies, as a consequence of damaging germline variants in single genes. Though initially identified among patients with unusual, severe or recurrent infections, non-infectious manifestations and especially immune dysregulation in the form of autoimmunity or autoinflammation can be the first or dominant phenotypic aspect of IEIs. An increasing number of IEIs causing autoimmunity or autoinflammation, including rheumatic disease have been reported over the last decade. Despite their rarity, identification of those disorders provided insight into the pathomechanisms of immune dysregulation, which may be relevant for understanding the pathogenesis of systemic rheumatic disorders. In this review, we present novel IEIs primarily causing autoimmunity or autoinflammation along with their pathogenic mechanisms. In addition, we explore the likely pathophysiological and clinical relevance of IEIs in systemic rheumatic disorders.

## Introduction

From a historical perspective, primary immune deficiencies (PIDs) have been primarily considered disorders of increased susceptibility to infections.^1^ Consistently, the first phenotypic descriptions of PIDs in 1950s referred to severe hereditary neutropenia, agammaglobulinemia and severe combined immunodeficiency (SCID).^1^^,^^2^ Subsequently, it became clear that malignancies and immune dysregulation belong to the phenotypic spectrum of PIDs and that in fact, non-infectious manifestations can be the main or even the initial clinical feature of PIDs.^1^^,^^3^ The latter together with the increasing recognition of the genetic basis of PIDs led to the term “inborn errors of immunity” (IEI),^1^ introduced in analogy to the definition of the “inborn errors of metabolism” by Archibald Edward Garrod.^4^

The broad availability of next generation sequencing (NGS) led to the identification of an increasing number of genetic defects falling under IEIs, whose number according to the current classification update by the International Union of Immunological Societies (IUIS) expert committee amounts to 485 disorders.^5^ Based on their phenotypes, IEIs are currently classified into 10 groups (or “tables”) of disorders, including the group of the phenocopies of IEIs. Among those, diseases of immune dysregulation (group IV) and autoinflammatory disorders (group VII) especially display phenotypic overlap with rheumatic disorders, though autoimmune and inflammatory manifestations are also common in disorders classified under alternative groups, such as combined immunodeficiencies or complement deficiencies.

The genetic components of rheumatic disorders have been explored based on the hypothesis-free approach of genome-wide association studies (GWAS), which primarily aim at detecting relatively common variants at a genome-wide level in large populations.^6^, ^7^, ^8^, ^9^ On the other hand, genetic studies in the considerably rarer IEIs, have focused on rare variants, taking advantage of NGS technologies.^5^^,^^10^ Either way, the investigation of genetics of IEIs and rheumatic disorders suggested overlapping genetic backgrounds by identifying a considerable proportion of risk genes in rheumatic disorders as disease-causing in monogenic IEIs.^11^^,^^12^

According to the German national registry of PIDs, 6% of PID diagnoses, fell under disorders of autoimmunity or immune dysregulation, whereas 25% of patients with PID displayed features of immune dysregulation, which was second most prevalent manifestation after infections.^13^ Similar data were obtained from the French and the South African registries, where autoimmune features were ranked second after infections.^14^^,^^15^ Evaluation of the prevalence of different forms of autoimmunity in the French registry, identified rheumatic disorders in approximately 13% of patients with autoimmune features.^14^ Inflammatory arthritis in particular has been commonly reported as a rheumatic feature of IEIs.^15^^,^^16^ Given the historical perception of IEIs as disorders characterized by susceptibility to infections, the abovementioned numbers may be based on cohorts biased towards patients with clinically evident immunodeficiency, underestimating the prevalence of rheumatic disease in IEIs.

As per definition, the term IEI implies the monogenic etiology of an immunological disorder. However, the incomplete penetrance as well as the variable expressivity of mutations that cause relatively common inborn errors of immunity, such as CTLA-4 insufficiency or STAT3 gain-of-function,^17^^,^^18^ question the strictly monogenic etiology of immune dysregulation in these disorders, suggesting the role of additional genetic and/or epigenetic modifiers as well as environmental factors. The biological consequences of genetic variants in a single gene may vary, depending on the type, the localization of the variant and the residual gene function. For example, female *CYBB* mutation carriers of CGD could develop systemic lupus erythematosus (SLE)-like disease, including photosensitive rash and aphthous ulcers, as a consequence of Lyonization, that can cause variable loss of gp91^phox^ function.^19^ Further, the impact of loss-of-function variants can vary from amorphic to hypomorphic with variable residual function and hypomorphic variants may account for milder disease and/or a latter disease onset.^20^ In addition, genes identified through GWAS to confer risk for rheumatic diseases overlap with genes associated with IEIs. In case of the rheumatoid arthritis (RA), 20 out of 152 non-MHC susceptibility loci are located in genes linked with IEI.^5^^,^^6^ All the aforementioned points suggest that the concept of a continuum between monogenic and polygenic immune dysregulation is more realistic than the strict division between monogenic and polygenic disorders.^21^

Nonetheless, the central etiopathogenic role of single genes in IEIs highlights molecular defects that can break immune tolerance. Therefore, identification of the genes and the pathways accounting for immune dysregulation in IEIs may aid understanding of the pathophysiology of autoimmunity and autoinflammation in the context of rheumatic disorders and lead to the identification of novel therapeutic targets to treat polygenic immune dysregulation. Others and we have previously reviewed the main mechanisms breaking immune tolerance in IEIs.^11^^,^^12^ Those include lymphopenia (e.g. RAG deficiency and Omenn syndrome), apoptosis defects (e.g. autoimmune lymphoproliferative syndrome (ALPS) and ALPS-like disorders), ineffective central tolerance [e.g. autoimmune polyendocrinopathy, candidiasis and ectodermal dystrophy (APECED), DiGeorge syndrome], impaired regulatory T cell (Treg) differentiation and/or function (e.g. IPEX and CTLA-4 insufficiency), complement defects (e.g. C1q deficiency, C4 deficiency), increased type I interferon production and/or signaling [e.g. Aicardi-Goutières syndrome (AGS)], aberrations in T cell receptor signaling [e.g. activated phosphoinositide 3-kinase δ syndrome (APDS)] and B cell-intrinsic defects [e.g. protein kinase C δ (PKCδ) deficiency, activation induced cytidine deaminase (AID) deficiency]. In this review, we aim at providing an update on the mechanisms that break immune tolerance in IEIs, focusing on the ones added in the current IUIS classification,^5^ whose pathogenesis is most relevant to systemic rheumatic disorders. In addition, we explore the practical relevance of the identification of IEI among patients with well-classifiable rheumatic diseases, typically diagnosed and managed by rheumatologists, such as inflammatory arthropathies, vasculitis and connective tissue diseases.

## Association of inborn errors of immunity with rheumatic disease

Several lines of evidence suggest rheumatic disease as a manifestation of IEIs. Sequencing projects on cohorts of patients with IEIs revealed that monogenic disorders are more commonly diagnosed among patients with manifest immune dysregulation, including organ-specific autoimmunity and arthritis.^22^, ^23^ Phenotypic characterization of patients with relatively common autosomal dominant disorders, such as the signal transducer and activator of transcription 3 (STAT3) gain-of-function, the cytotoxic T-lymphocyte-associated protein 4 (CTLA-4) insufficiency or heterozygous loss-of-function variants in *NFKB1*, revealed rheumatic manifestations, more commonly in the form of arthritis or vasculitis in considerable proportion of patients, exceeding 10% in each of the aforementioned monogenic disorders.^17^^,^^18^^,^^24^ Vise versa, patients with well classifiable rheumatic disease, such as RA, juvenile idiopathic arthritis (JIA) or psoriatic arthritis (PsA) as their main or sole manifestation, were diagnosed with an underlying IEI, suggesting that IEIs can manifest as typical rheumatic disorders. Further, given the fact that the majority of patients subjected to genetic testing for IEIs come to attention due to their immunodeficiency, i.e. their recurrent infections, may result in the underestimation of the prevalence of rheumatic disease in IEIs and especially of the proportion of patients with a rheumatic disease as their only manifestation.

## Update on the mechanisms of autoimmunity and autoinflammation in inborn errors of immunity

### The role of dysregulated innate immune sensing

Innate immune sensing of pathogen associated molecular patterns (PAMPs) stimulates B cells, T cells and antigen presenting cells.^25^^,^^26^ Similar to PAMPs, endogenous molecules, commonly released from dying cells, falling under damage-associated molecular patterns (DAMPs), can stimulate immune cells. Overall, innate immune sensing of PAMPs and DAMPs has a proinflammatory effect that may be relevant for the pathogenesis of rheumatic disorders, such as for RA and SLE.

The E74 Like ETS transcription factor 4 (ELF4) is a member of the E26 transformation-specific (ETS) transcription factors, that is ubiquitously expressed by immune cells, including T cells, NK cells and myeloid cells.^27^ Hemizygous loss-of-function mutations in *ELF4* have been recently reported to result in systemic autoinflammation in 4 patients, manifesting with arthritis, colitis, Behçet's-like orals ulcers and SLE-like rash.^28^^,^^29^ Studies on Elf4 deficient and knock-in mice harboring loss-of-function mutations, have revealed several roles for Elf4 in the immune system, including its involvement in NK and NKT cell development and function, in CD8-T cell activation and differentiation, in Th17 differentiation of helper T cells as well as in antiviral type I interferon (IFN)-mediated immunity and in regulating Toll-like receptor (TLR) 4 signaling. However, inflammatory manifestation have not been reported in Elf4-deficient mice housed under specific pathogen-free conditions.^30^ The latter together with immunological findings and the infection-exacerbated disease course, suggest dysregulated innate immune sensing and the consequent overproduction of proinflammatory cytokines as the mechanism of systemic autoinflammation in patients with hemizygote loss-of-function mutations in *ELF4*.^29^ Ineffective handling of infectious agents, especially viruses,^31^ may be also relevant for enhanced innate immune activation in ELF4 deficiency.

The spleen tyrosine kinase (SYK) is an immunoreceptor-associated kinase with tandem SH2 domains, which is primarily expressed in mononuclear phagocytes, epithelial cells and B cells.^32^ SYK is involved in signaling downstream of the B cell receptor (BCR), the Fc receptors, integrins and the TLRs. Monoallelic gain-of-function variants in *SYK* result in systemic inflammation including arthritis, eczema, vasculitis and colitis, several aspects of which were recapitulated in knock-in mice.^33^ Identified patients displayed increased frequencies of Th17 and Th1 cells. Joint inflammation in knock-in mice was dominated by macrophage and T cell infiltrates, which given the role of SYK in monocytes and macrophages may suggest innate immune hyperactivation through enhanced integrin and TLR signaling. The absence of spontaneous intestinal inflammation in knock-in mice together with the enhanced production of proinflammatory cytokines by epithelial cells after their stimulation with zymosan, suggest the pathogenic role of innate immune sensing, likely through TLRs, at least in SYK gain-of-function-associated colitis.

TLR7 recognizes single-stranded RNA.^34^ GWAS have associated *TLR7* polymorphisms with SLE and recently gain-of-function mutations in *TLR7* have been reported to cause a monogenic form of SLE.^35^ Mechanistically, those mutations enhanced affinity to guanosine, thereby lowering the activation threshold of TLR7.^36^ Similar to TLR7, human TLR8 senses single-stranded RNA.^37^ Its transgenic expression in mice, resulted in systemic inflammation, including autoimmune pancreatitis, hepatitis and spontaneous arthritis, suggesting the strong proinflammatory effect of TLR8 signaling.^37^ Human germline or mosaic gain-of-function variants in *TLR8* have been associated with neutropenia, hypogammaglobulinemia, lymphoproliferation, autoimmunity and bone marrow failure.^38^ Peripheral blood analysis in these patients revealed high serum levels of proinflammatory cytokines, including interleukin (IL)-18 and IFN-γ as well as a type I IFN signature.

### Defects enhancing immunogenicity of self-DNA cause type I interferonopathies through the activation of the cGAS-STING pathway

Abnormal induction of type I IFNs results in a group of disorders, the type I interferonopathies, that typically manifest with vasculopathy, myositis or early-onset SLE, the prototype of which is AGS.^12^ The genetic spectrum of type I interferonopathies includes genes encoding nucleases, innate immune sensors of nucleic acids and associating signaling mediators as well as proteasome-related proteins.

The stimulator of IFN response cGAMP interactor 1 (STING), previously known as transmembrane protein 173 (TMEM173), is a signaling mediator involved in sensing of double-stranded DNA (dsDNA) from intracellular pathogens by the cyclic GMP-AMP synthase (cGAS), which leads to the induction of type I IFNs.^39^ Both heterozygous and homozygous *STING* mutations can hyperactivate STING and cause the STING-associated vasculopathy with onset in infancy (SAVI), i.e. a monogenic type I interferonopathy, characterized by interstitial lung disease (ILD), cutaneous vasculitis and arthritis, that typically has a very early-onset in infancy.^17^^,^^40^

COPA syndrome is an autosomal dominant disorder characterized by inflammatory lung disease, arthritis and kidney disease.^41^^,^^42^ It is caused by pathogenic variants in the coatomer protein complex subunit alpha (*COPA*) gene that encodes the coatomer protein required for cytoplasmatic trafficking between the endoplasmic reticulum (ER) and the Golgi apparatus. Increased ER stress due to impaired cellular trafficking and aberrant autophagy have been proposed to account for autoinflammation in COPA syndrome. However, the recent identification of the cGAC-STING-mediated overproduction of type I interferons, suggests a shared pathogenesis of COPA syndrome and SAVI, which would be in accordance with the phenotypic overlap between those autoinflammatory IEIs.^43^

Biallelic loss-of-function mutations in *RNU7-*1 and *LSM11* have been reported to cause an autosomal recessive subtype of AGS through the activation STING.^44^ *RNU7-1* encodes the small nuclear RNA (snRNA) U7, whereas *LSM11* encodes the U7 snRNA-associated Sm-like protein (LSM11), which is part of the U7 snRNP complex. The latter is involved in processing of the replication-dependent histone (RDH) pre-mRNA. Defects in processing of RDH pre-mRNA and consequently disturbed linker histone stoichiometry have been suggested to result in aberrant interferon signaling through the activation of cGAS and consequently STING.

The ATPase family AAA domain-containing protein 3A (ATAD3A) is a ubiquitously expressed mitochondrial protein involved in the maintenance of mitochondrial DNA (mtDNA).^45^ Dominant-negative heterozygous mutations in *ATAD3A* has been shown to result in a type I interferonopathy including systemic sclerosis-like disease.^46^ Mechanistically, reduced activity of ATAD3A has been proposed to result in enhanced type I IFN signaling through the cGAS-STING pathway in a cytosolic mtDNA-dependent manner, as shown after knockdown of ATAD3A in a monocyte cell line as well as in fibroblasts from patients with *ATAD3A* mutations. These finding suggest the immunogenicity of cytosolic mtDNA as a mechanism of type I interferonopathy.

### Dysregulated cellular stress responses can induce the production of type I interferons

The β5i subunit is a component of the immunoproteasome with protease activity encoded by the proteasome subunit beta type-8 gene (*PSMB8*).^47^ On cytokine-induced activation of hematopoietic cells and fibroblasts, the β5i subunit substitutes the β5 subunit of the constitutive proteasome.^48^ Variants in *PSMB8* gene have been associated with autoinflammatory disorders, such as the Nakajo-Nishimura syndrome and the chronic atypical neutrophilic dermatosis with elevated temperature (CANDLE).^47^ Biallelic mutations affecting other subunits of the proteasome result in autoinflammatory disease, classified as a proteasome-associated autoinflammatory syndrome (PRAAS).

The proteasome subunit beta type-9 gene (*PSMB9*) encodes the β1i subunit of the immunoproteasome, whose expression is induced in cytokine-stimulated hematopoietic cells and fibroblasts.^48^ Further, β1i is a subunit of the thymoproteasome, expressed by cortical epithelial cells of the thymus. Three unrelated patients with a heterozygous missense variant in *PSMB9* (G156D), developed severe infantile-onset PRAAS-like autoinflammation, manifesting with fever, eczemas, juvenile dermatomyositis and basal ganglia calcification, associating with hyperactivation of IFN-α.^49^^,^^50^ Similar to other proteasome-related IEIs, enhanced interferon production in patients with the G156D variation in *PSMB9* variant may be stemming from the accumulation of polyubiquitylated proteins and the consequently induced ER-stress.^51^ These patients in addition displayed combined immunodeficiency, which deviated from the phenotype of PRAAS and which was recapitulated by knock in mice harboring same monoallelic mutation. However, Psmb9^G156D/+^ mice displayed no spontaneous autoinflammation.^51^ The authors suggest that not yet defined environmental factors may be required for autoinflammation.

The ubiquitin-activating enzyme 1 (UBA1), encoded from the homonymous gene, that lies in the X chromosome, is the major enzyme for the initiation of ubiquitylation.^52^^,^^53^ Ubiquitylation is a post-translational modification that is relevant for several aspects of cell biology, such as the proteasomal degradation of proteins, cell cycle progression, the regulation of autophagy and innate immune signaling. Somatic mutations in *UBA1*, have been reported to cause VEXAS (vacuoles, E1 enzyme, X-linked, autoinflammatory, somatic) syndrome, a severe inflammatory disorder characterized by a heterogeneous clinical spectrum that includes fever, cytopenias, dysplastic bone marrow with characteristic vacuoles in the erythroid and myeloid precursors, cutaneous and pulmonary inflammation, chondritis and various types of vasculitis. Patients diagnosed with VEXAS commonly fulfill clinical criteria for inflammatory disorders, such as relapsing polychondritis, polyarteriitis nodosa or hematologic diseases, i.e. multiple myeloma or myelodysplastic syndrome.

Somatic mutations in *UBA1* that cause VEXAS are located at the methionine 41 (M41) residue or close to it and abrogate the expression of the short isoform of UBA1 (UBA1b), which starts with M41 and is localized in the cytoplasm, in contrast to its longer counterpart (UBA1a) which mainly displays a nuclear localization.^53^ The loss of cytoplasmic expression of UBA1 and the consequently decreased ubiquitylation have been suggested to trigger cellular stress and in particular the unfolded protein response (UPR), which induces the production of type I IFNs.^54^ Inflammatory signatures in peripheral blood of patients with VEXAS are consistent with the activation of the UPR.^52^

### Hyperactivation of myeloid cells

The *CEBPE* gene encodes the CCAAT enhancer-binding protein ε (C/EBPε), which is a transcription factor involved in differentiation and function of myeloid cells.^55^ Loss-of-function variants in CEBPE have been linked with the neutrophil-specific granule deficiency.^56^ The homozygous R219H variant has been identified in a family and reported to cause an autosomal recessive disorder characterized by autoinflammation (pyoderma gangrenosum, granulomas, aseptic fever) and immunodeficiency due to neutrophil dysfunction, i.e. the C/EBPε-associated autoinflammatory and immune impairment of neutrophils (CAIN).^57^ Mechanistically, the homozygous Arg219His variant has been shown to both induce the noncanonical activation of the inflammasome in monocytes and macrophages and enhance IFN responses in neutrophils.

Hematopoietic cell kinase (HCK) is a highly conserved member of the Src kinases involved in neutrophil and macrophage effector functions, such as phagocytosis and innate immune sensing.^58^ Heterozygous gain-of-function mutations in *HCK* resulted in increased kinase activity of HCK and enhanced activation of myeloid cells, including migration and production of inflammatory cytokines [IL-1β, IL-6, IL-8, tumor necrosis factor (TNF)-α] resulting in an autoinflammatory disorder characterized by cutaneous vasculitis and interstitial lung disease.^59^

### Impaired regulatory T cell differentiation and function

Tregs are immunosuppressive T cells that play a dominant role in the regulation of immune responses.^11^ Treg dysfunction has been associated with a variety of rheumatic disorders, including RA, SLE, systemic sclerosis and giant cell arteritis.^60^^,^^61^ Severe immune dysregulation affecting multiple organs in IEI causing Treg deficiency or dysfunction, highlights their significance in the maintenance of immune tolerance.^11^^,^^12^ The prototype IEI due to loss of Tregs is the immunodysregulation, polyendocrinopathy, enteropathy, X-linked syndrome (IPEX), which is caused by biallelic loss-of-function mutations in *FOXP3*. The spectrum of IEI characterized by immune dysregulation associated with Treg defects includes 10 genes, according to the current IUIS classification (*FOXP3*, *CTLA4*, *LRBA*, *DEF6*, *IL2RA*, *IL2RB*, *STAT3*, *BACH2*, *FERMT1* and *IKZF1*),^5^ though additional genes, such as *STAT5b*, *PIK3CD* and *DOCK8* have been associated with Treg dysfunction.^11^

The transcription factor Ikaros, encoded by the gene *IKZF1*, is involved in hematopoiesis and lymphocyte development.^62^ Recently, heterozygous gain-of-function genetic variants in *IKZF1* have been identified to cause a disorder characterized by IPEX-like immune dysregulation, including autoimmunity in the form of autoimmune cytopenias, vitiligo, type 1 diabetes, Hashimoto's thyroiditis and autoimmune hepatitis as well as gastrointestinal disease (inflammatory colitis and celiac disease), atopy and benign lymphoproliferation.^63^ Besides an IPEX-like phenotype, immune dysregulation in Ikaros gain-of-function associated with reduced production of IL-2, reduced Treg counts and impaired Treg differentiation. Similar to Ikaros gain-of-function, loss-of-function, especially in case of haploinsufficiency, can also cause autoimmunity, in the form of autoimmune cytopenias, SLE, antiphospholipid syndrome (APS) and myasthenia gravis, which however, tends to localize at a single organ and is rather attributed to B cell dysregulation.^64^^,^^65^

*IKZF2* encodes Helios, a member of the Ikaros family of transcription factors.^66^ Helios is known to control lymphocytes development and the differentiation of NK cells and T follicular helper cells. In case of Tregs, Helios has been shown to stabilize FOXP3 expression, thereby maintaining their suppressive potential.^67^ Germline monoallelic or biallelic loss-of-function variants in *IKZF2* have been reported to cause a disorder characterized by combined immunodeficiency, Epstein–Barr virus (EBV)-associated complications and immune dysregulation.^68^, ^69^, ^70^ The latter includes benign lymphoproliferation, SLE and autoimmune cytopenias. Mechanistically, identified pathogenic variants have been shown to affect the interaction of Helios with its Ikaros family counterparts, its binding to members of the nucleosome remodeling deacetylase (NuRD) chromatin remodeling complex and/or cause reduced Helios expression, which has been shown to associate with increased production of proinflammatory cytokines by effector T cells and Tregs.^68^, ^69^, ^70^ Therefore, in addition to Treg dysfunction, Helios loss-of-function-associated immune dysregulation relates to failed control of EBV and enhanced expression of proinflammatory cytokines.

### Inadequate expression of co-inhibitory molecules

The outcome of the recognition of epitopes presented by major histocompatibility complex (MHC) molecules by the T cell receptor (TCR) depends on co-stimulatory and co-inhibitory receptors and their ligands.^11^^,^^12^ The cytotoxic T-lymphocyte-associated protein 4 (CTLA-4) is a co-inhibitory molecule, expressed on Tregs and activated conventional T cells and functions through antagonizing the costimulatory interaction of CD28 with CD80 and CD86.^11^ Monoallelic mutations in *CTLA4* resulting in inadequate expression of CTLA-4 lead to a disorder characterized by variable immunodeficiency and immune dysregulation.^18^ The latter commonly manifests with benign lymphoproliferation, enteropathy, autoimmune cytopenias, eczemas and arthritis. Programmed death-1 (PD-1), like CTLA-4 a co-inhibitory receptor, that negatively regulates T cell activation.^71^ Unlike CTLA-4, PD-1 displays a broader expression pattern, being mainly expressed by B cells, T cells and NK cells as well as monocytes, macrophages and dendritic cells, and is induced at a later stage after T cell activation.^71^^,^^72^ PD-1 interacts with the programmed death-ligand (PD-L) 1, which is expressed on various immune cells and a variety of tissues, such as the lungs, the liver and the kidneys as well as with PD-L2, whose expression is restricted to macrophages and dendritic cells. Consistent with the inhibitory role of PD-1 on T cell activation, PD-1 deficient mice are prone to severe SLE-like disease after the introduction of the *lpr* mutation, lethal autoimmune pancreatitis, in case of reduced FOXP3 expression and severe collagen-induced arthritis.^72^, ^73^, ^74^, ^75^ Human PD-1 deficiency, is a recently identified monogenic disorder, mainly characterized by immune dysregulation, that manifests with polyautoimmunity, including endocrinopathies and JIA,^76^ that resemble the likely side-effects of PD-1 blockade to treat cancer.^77^

### Regulation of cytokine signaling

The suppressors of cytokine signaling (SOCS) are a family of proteins involved in negative feedback regulation of JAK-STAT signaling.^78^ SOCS1 primary regulates signaling of IFNs and cytokines of the IL-2 family. It directly binds and inhibits the Janus kinase (JAK) 1, JAK2 and the tyrosine kinase 2 (TYK2), preventing the phosphorylation of signal transducer and activator of transcription (STAT) 1 and STAT2. In addition, SOCS1 is involved in TCR and TLR signaling. Mice lacking SOCS1 display severe immune dysregulation due to uncontrolled activation of type I and II IFNs.^79^ In addition, SOCS1 haploinsufficiency in mice results in SLE-like disease.^80^ Human SOCS1 haploinsufficiency has been reported to cause early-onset benign lymphoproliferation and autoimmunity, manifesting with autoimmune cytopenias, thyroidopathy, arthritis, psoriasis and SLE,^81^, ^82^, ^83^ resembling immune dysregulation observed in other IEI, such as STAT1 gain-of-function.^84^

STAT2 has been shown to regulate type I IFNs.^85^ A biallelic loss-of-function variant in *STAT2* (p. Arg148Trp) has been shown to cause a type I interferonopathy, characterized by neurological damage, including intracranial calcification and systemic inflammation. The consequences of this mutation deviate from STAT2 deficiency, which results in susceptibility to viral infections.^86^ Mechanistically, mutant STAT2 (R148W) fails to interact with the ubiquitin-specific protease 18 (USP18), resulting in enhanced IFNα/β signaling. USP18 is a negative regulator of IFNα/β signaling and its deficiency results in a type I interferonopathy.^87^ STAT2 acts as an adaptor for recruiting USP18 to the interferon-receptor 2 subunit (IFNAR2).^88^ Recruitment of USP18 negatively regulates receptor dimerization, binding of interferon and JAK1 phosphorylation.

### Autoimmune lymphoproliferative syndrome and aberrations in cell death

ALPS is characterized by chronic benign lymphoproliferation, autoimmunity, including autoimmune cytopenias and SLE-like disease as well as increased lymphoma risk attributed to defects in lymphocyte apoptosis.^89^ Besides defects affecting Fas-mediated extrinsic apoptosis (mutations in *FAS* (encoding Fas), *FASLG* (encoding Fas ligand) and *CASP10* (encoding caspase 10)),^90^ more than 10 monogenic disorders have been reported to cause ALPS-like phenotypes.^89^ Among those *NRAS* and *KRAS* mutations cause apoptosis defects as well, though they affect the intrinsic apoptosis pathway.^11^^,^^89^^,^^90^ The genetic spectrum of ALPS has been recently expanded through the identification of biallelic germline loss-of-function variants in TET2, in patients with immunodeficiency, benign lymphoproliferation, autoimmune cytopenias and lymphomas.^91^^,^^92^ The ten-eleven translocation methylcytosine dioxygenase 2 (TET2) is a 2-oxoglutarate- and Fe^2+^-dependent enzyme of the ten-eleven translocation methylcytosine dioxygenase (TET) family of epigenetic regulators. TET2 is involved in DNA demethylation by catalyzing the conversion of 5-methylcytosine into 5-hydroxymethylcytosine. Besides an ALPS phenotype, patients with TET2 loss-of-function fulfilled several laboratory criteria of ALPS, including increased proportion of double-negative T cells (three out of five reported patients) and impaired Fas-dependent apoptosis (two out of four tested patients). Loss of TET2 function results in increased blood DNA methylation levels, especially affecting active enhancers and regulatory regions involved in the regulation of hematopoietic differentiation. Tested patients displayed aberrant B cell differentiation with reduced class-switched memory B cells as well as skewing of T cell differentiation towards Th2 cells. Co-segregation of autoimmunity and impaired T-cell apoptosis may suggest the pathogenic role of an apoptosis defect in development of manifest autoimmunity in patients with TET2 loss-of-function. Further, like in ALPS,^93^ impairment of apoptosis may also lead to the accumulation of autoreactive B cells. However, the latter as well as the exact involvement of TET2 in apoptosis need to be further investigated. As Tregs from Tet deficient mice displayed abnormal proliferation along with FOXP3 destabilization,^94^ Treg dysfunction may represent an additional mechanism of autoimmunity in patients with TET2 defect. As for lymphoproliferation, besides impaired apoptosis, TET2 loss-of-function may alternatively or in addition promote lymphocyte proliferation. Finally, similar to other IEIs characterized by failed control of EBV, such as the IL-2-inducible T Cell kinase (ITK) deficiency or the X-linked immunodeficiency with magnesium defect, EBV infection and neoplasia (XMEN) syndrome, autoimmunity in TET2 deficiency manifests primarily with autoimmune cytopenias.^11^ Given the latter and the connection between EBV and autoimmunity, it can be speculated that failed control of EBV and EBV-driven mechanisms might be involved in TET2 deficiency-associated immune dysregulation.

Unlike the immunologically quiescent nature of traditional apoptosis, alternative modes of cell death, such as necrosis and necroptosis are immunogenic through the release of damage-associated molecular patterns (DAMPs) and may be involved in the pathogenesis of inflammatory disorders.^95^^,^^96^ The receptor-interacting serine/threonine-protein kinase 1 (RIPK1) modulates innate immunity by directing innate immune signaling towards pro-survival and pro-inflammatory responses or caspase-8-mediated apoptosis.^97^ RIPK1 is regulated through post-translational modification, including phosphorylation and ubiquitination. Heterozygous mutations affecting the residue aspartic acid 324 (D324) of RIPK1, result in an early-onset autoinflammatory disorder characterized by recurrent fever and lymphoproliferation.^98^^,^^99^ Mechanistically, replacement of the D324 residue, affects cleavage of RIPK1 by caspase-8, leading to RIPK1 overactivation and autophosphorylation promoting RIPK1-dependent cell death and necroptosis. In contrast to monoallelic mutations replacing the D324 residue, biallelic loss-of-function mutations in *RIPK1* result in severe early-onset immunodeficiency with lymphopenia, recurrent viral, bacterial and fungal infections.^100^^,^^101^ In addition, patients display inflammatory disease, such as inflammatory bowel disease and polyarthritis, whose pathogenesis has not been elucidated yet. However, association of inflammation with enhanced production of IL-1β in patients with biallelic loss-of-function mutations in *RIPK1* may suggest hyperactivation of NLPR3.

TBK1, referred here previously in the context of the cGAS-STING signaling,^102^ is a serine/threonine protein kinase of the IkappaB kinase (IKK) family, involved in antiviral immunity, type I IFN and pro-inflammatory cytokine production.^103^^,^^104^ In addition, TBK1 regulates the early stages of autophagy as well as the TNF-induced RIPK1-dependent cell death.^105^, ^106^, ^107^ Homozygous loss-of-function mutations in *TBK1* result primarily in a systemic autoinflammatory disorder characterized by variable neurological damage, arthritis and vasculitis.^108^ Mechanistically, autoinflammation can be at least in part explained through the enhanced TNF-induced RIPK1-dependent cell death, as shown in fibroblasts from patients with TBK1 loss-of-function and suggested by the therapeutic efficacy of TNF inhibitors in these patients.

### Multifactorial immune dysregulation

**Fig. 1:**
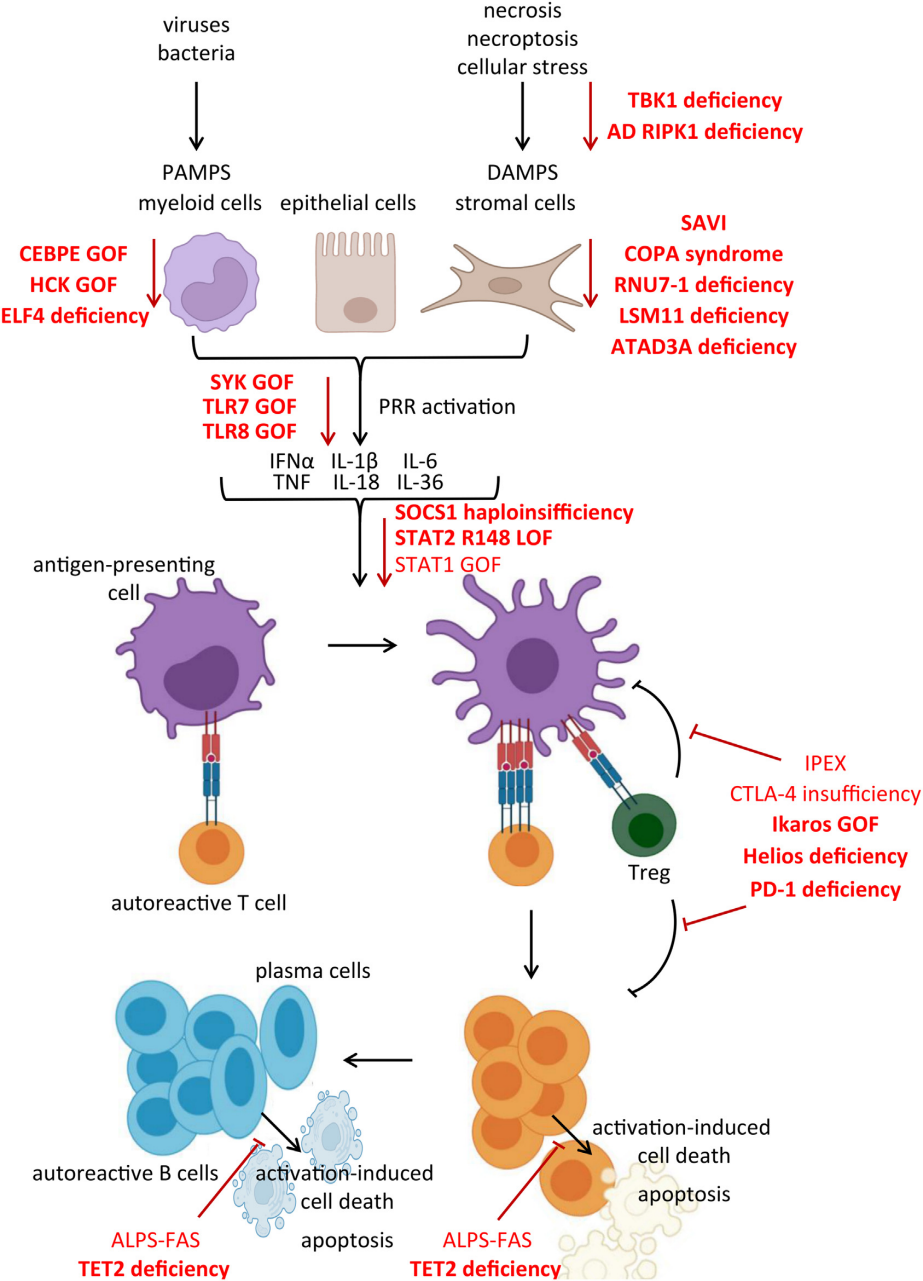
Summary of the mechanisms that break immune tolerance in novel inborn errors of immunity (marked with bold characters) of groups IV and VII added in the 2022 IUIS classification. Those include enhanced proinflammatory cell death [TBK1 deficiency, autosomal dominant (AD) RIPK1 deficiency], the activation of myeloid cells [CEBPE gain-of-function (GOF), HCK GOF], the activation of the cGAS-STING pathway (SAVI, COPA, RNU7-1 deficiency, LSM11 deficiency), the hyperactivation of innate immune receptors (SYK GOF, TLR7 GOG, TLR8 GOF), the dysregulated signaling of proinflammatory cytokines (SOCS1 haploinsufficiency, STAT2 R148 LOF, STAT1 GOF), defects in the differentiation and/or function of Tregs (Ikaros GOF, Helios deficiency) and defects in apoptosis of activated lymphocytes (ALPS, TET2 deficiency). ALPS, autoimmune lymphoproliferative syndrome; AD, autosomal dominant; CEBPE, CCAAT enhancer-binding protein ε; cGAS, cyclic GMP-AMP synthase; COPA, coatomer protein complex subunit alpha; GOF, gain-of-function; CTLA-4, cytotoxic T-lymphocyte-associated Protein 4; DAMPS, damage-associated molecular patterns; HCK, hematopoietic cell kinase; IFN, interferon; IL, interleukin; IPEX, Immunodysregulation polyendocrinopathy enteropathy X-linked; IUIS, International Union of Immunological Societies; PAMPS, pathogen-associated molecular patterns; PRR, pattern recognition receptor; RIPK1, receptor-interacting serine/threonine-protein kinase 1; SAVI, STING-associated vasculopathy with onset in infancy; SOCS, suppressors of cytokine signaling; STING, stimulator of IFN response cGAMP interactor 1; SYK, spleen tyrosine kinase; TAT, signal transducer and activator of transcription; TET2, ten-eleven translocation methylcytosine dioxygenase 2; TLR, Toll-like receptor; TNF, tumor necrosis factor; Tregs, regulatory T cells; R, arginine.

Evaluation of the existing literature on functional consequences of novel genetic defects, provided in most cases at least one pathophysiological correlate of immune dysregulation (Fig. 1). On this basis, we aspired to a pathophysiological classification of the mechanisms of immune dysregulation in IEIs, which is by no means perfect. Especially the pleiotropic consequences of previously discussed defects affecting transcription factors (e.g. Helios and C/EBPε) or epigenetic modifications (e.g. TET2), are expected to result in immune dysregulation through more than one pathomechanisms. The hematopoietic protein 1 (HEM1) deficiency^109^ and the NEMO exon 5 deletion^110^ exemplify IEIs that cause immune dysregulation by affecting diverse levels of the immune tolerance.

HEM1, alternatively called Nck-associated protein 1-like (NCKAP1L), encodes a hematopoietic lineage-restricted member of the nucleosome assembly protein 1-like (Nap1l) subunit of the Wiskott-Aldrich syndrome protein (WASP)-family verprolin-homologous protein (WAVE), which is a key component of the machinery of the actin cytoskeleton.^111^^,^^112^ Together with the Rho GTPase Rac, HEM-1 promotes branched actin polymerization through the Arp2/3 complex, in response to the activation of various immune receptors, including the B cell receptor (BCR), the TCR, cytokine receptors and TLRs. Hem1 deficient mice have established the role of Nckap1L in the activation, differentiation, adhesion and migration of T cells as well as for the function of neutrophils.^113^ More recently, HEM-1 has been shown to regulate B cell development through its involvement in BCR signaling.^109^ Human HEM1 deficiency as a consequence of homozygous loss-of-function mutations in *NCKAP1L* causes a syndrome of immunodeficiency characterized by variable immune dysregulation including autoimmunity, lymphoproliferation, features of hemophagocytic lymphohistiocytosis (HLH) and atopic disease.^109^^,^^111^^,^^114^

*IKBKG* encodes the NF-κB essential modulator (NEMO), a regulatory subunit of the IKK complex, which negatively regulates NF-κB activation.^115^ Variants in *IKBKG* resulting in deletion of exon 5 have been reported to cause an autoinflammatory disorder, named NEMO-deleted exon 5-autoinflammatory syndrome (NDAS), that is characterized by systemic inflammation with fever, panniculitis, granulomas, uveitis and sterile osteomyelitis.^110^ Autoinflammatory features and lack of severe infections distinguish NDAS from other disorders due to hypomorphic *IKBKG* variants impairing NEMO expression and/or function, that are characterized by ectodermal dysplasia and immunodeficiency.^116^ Loss of NEMO domain encoded by the exon 5, results in enhanced activation of NF-κB in fibroblasts, T cells and monocytes, and associates with an IFN and NF-κB signature in peripheral blood.^110^

## Inborn errors of immunity in patients diagnosed with rheumatic disorders

Several pieces of evidence suggest that a subset of patients diagnosed with rheumatic disorders may actually have a monogenic IEI. Those include the growing number of monogenic disorders of immune dysregulation and consequently, the increasing recognition of the fact that rheumatic manifestations may represent the main or even the only phenotype of IEIs, the overlapping genetic backgrounds between IEIs and rheumatic disorders and the relevance of the pathomechanisms of IEI-associated immune dysregulation in rheumatic disease.^11^ In particular, typical rheumatic disease can precede the onset of clinical features or laboratory findings of immunodeficiency, which may develop during treatment with disease-modifying antirheumatic drugs (DMARDs) and steroids.^3^^,^^117^ Given the variable expressivity, which is relatively common among disorders of immune dysregulation, such as the CTLA-4 insufficiency and the STAT3 gain-of-function,^13^^,^^14^ on several occasions, segregation analysis has revealed typical rheumatic disease without significant evidence of immunodeficiency to segregate with a deleterious mutation in families, where the index patient came to attention and was subjected to genetic sequencing due to their immunodeficiency.^118^^,^^119^ Investigation of the functional impact of genetic variants identified through GWAS to confer susceptibility for rheumatic disorders, reveal their significant contribution to the pathomechanisms of rheumatic diseases, which has been evaluated in relevant independent cellular or mouse models.^120^^,^^121^ Therefore, some of the genetic variations identified through GWAS, may be fulfilling the IUIS criteria that define an IEI.^5^ NGS projects in subgroups of patients with rheumatic disorders have led to the identification of IEI-associated mutations. In particular, NGS in a mixed cohort of rheumatic patients with secondary hypogammaglobulinemia, revealed that approximately half of tested patients harbored at least one variant in genes associated with IEIs, including 15% of tested patients who were identified with predicted pathogenic variants in genes linked to autosomal dominant IEI and 3% with a variant that has been previously reported in the literature as pathogenic in the context of monogenic IEIs.^117^ Further, in a cohort of patients with psoriatic arthritis, targeted NGS, aimed primarily at the identification of variants in genes associated with autoinflammatory disorders, revealed that approximately 16% of tested patients with PsA harbored a likely deleterious germline variation in genes such as *AP1S3*, *CARD14*, *COPA*, *PLCG2, NOD2*, *NLRP12* and *TNFAIP3* that could account for an autoinflammatory IEI.^122^ Finally, HLH represents an additional phenotypic overlap between IEIs and some rheumatic disorder.^123^ Familial (or primary) HLH syndromes fall under group IV disorders and typically present early in life.^5^ Rheumatic disorders, such as the systemic JIA, SLE and adult-onset Still's disease associate with HLH, classified as secondary HLH and in particular as macrophage activation syndrome (MAS).^124^ However, genetic studies in patients with MAS have revealed rare variants in familial HLH-associated genes, including (*LYST*, *MUNC13-4* and *STXBP2*) in more than a third of tested patents, questioning the dichotomous view of HLH as familial (primary) or secondary.^125^^,^^126^

### Clues to the diagnosis of an IEI in patients with rheumatic disorders

Given the clinical spectrum of IEI, the Jeffrey Modell Foundation's warning signs may be suggesting an underlying IEI in patients with rheumatic conditions.^127^ Consistently, severe, recurrent, persistent or unusual infections, especially in rheumatic patients without predisposing factors, including immunosuppressive regimens that may be accounting for a pathological infection record,^128^ could suggest an underlying IEI. The manifestation of immune dysregulation may deviate from diagnosed rheumatic disorder, especially relevant in patients with polyautoimmunity or treatment refractory inflammation, which may represent a sign of an IEI. In patients with articular, especially seronegative disease, extraarticular manifestations such as interstitial lung disease or inflammatory bowel disease may be ‘red flags’ suggesting an IEI. IEI-associated manifestations, such as autoimmune cytopenias, particular types of malignancies and sarcoid-like inflammation could be also providing clues to an underlying IEI. Given the genetic basis of IEIs, a history of familial immune dysregulation or immunodeficiency should be considered as a definite ‘red flag’ for an underlying IEI.

**Fig. 2:**
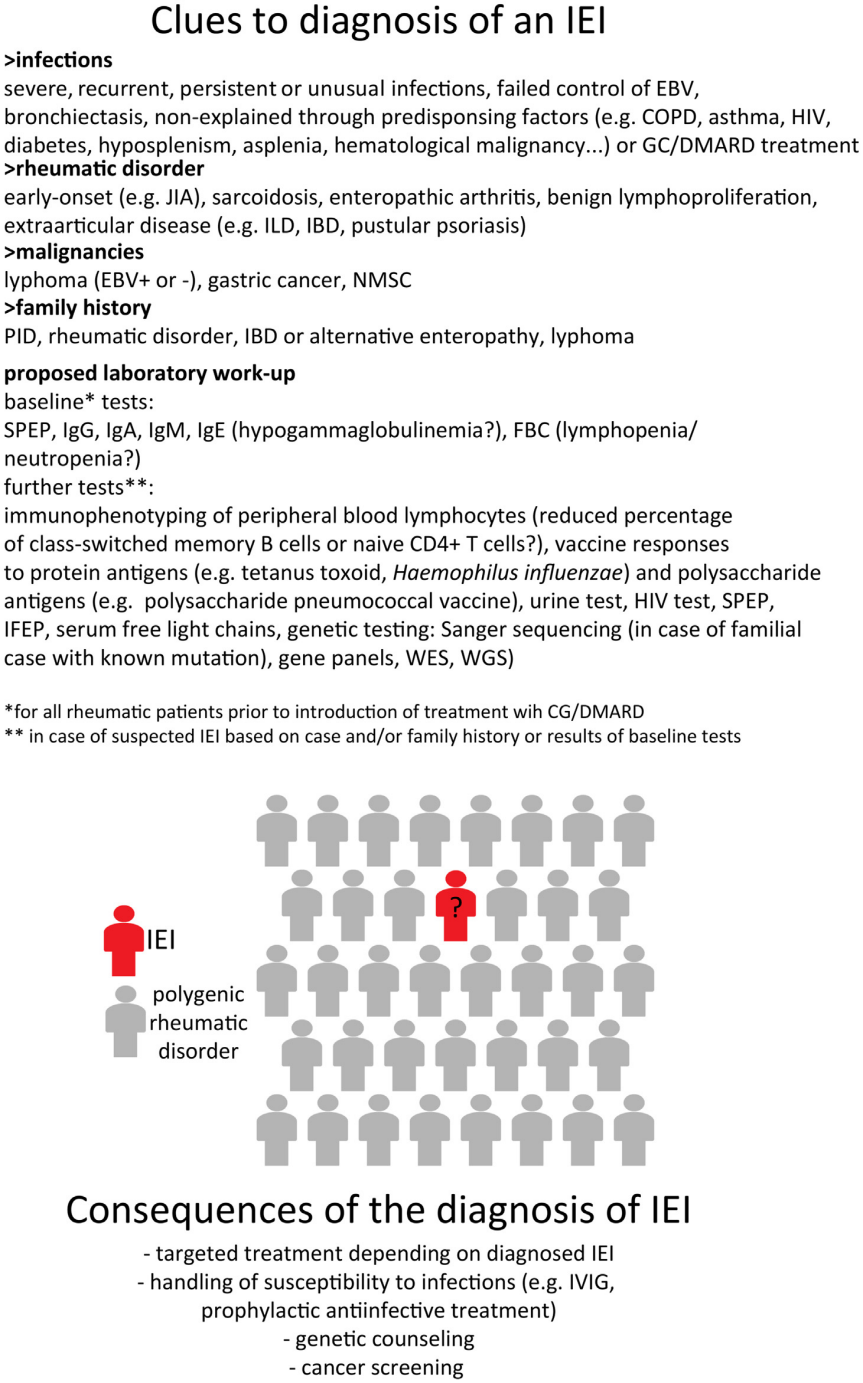
Inborn errors of immunity (IEIs) within rheumatic disorders. Likely clues that should raise suspicion for an underlying IEI among rheumatic patients, proposed immunological investigations and possible consequences from the diagnosis of an IEI in rheumatic patients. COPD, chronic obstructive pulmonary disease; DMARD, disease-modifying anti-rheumatic drug; EBV, Epstein–Barr virus; FBC, full blood count; GC, glucocorticoids; IFEP, immunofixation electrophoresis; IBD, inflammatory bowel disease; ILD, interstitial lung disease; JIA, juvenile idiopathic arthritis; HIV, human immunodeficiency virus; NMSC, non-melanoma skin cancer; PID, primary immunodeficiency disorder; SPEP, serum protein electrophoresis; WES, whole exome sequencing; WGS, whole genome sequencing.

In our opinion, laboratory tests aiming at evaluating an IEI in patients with a rheumatic condition should not differ from the ones employed in patients without a diagnosed rheumatic disease. Measurement of immunoglobulin levels and full blood count with white blood cell differential prior to the introduction as well as during immunosuppressive treatment can identify a preexisting immunodeficiency and differentiate it from a treatment-induced secondary immunodeficiency or a disease-activity-related cytopenia. In Fig. 2, we propose a set of immunological investigations that may be relevant for the diagnosis of an underlying IEI in patients with a well-classifiable rheumatic disorder. In our center, we routinely employ genetic testing for patients with a family history suggestive of an IEI, those with otherwise unexplained evidence of immunodeficiency or manifestations of immune dysregulation that deviate from the clinical spectrum of diagnosed rheumatic disorder. The identification of the prevalence of IEIs in each rheumatic disorder, the unbiased characterization of the penetrance of disease-causing variants and the natural history of each monogenic condition represent obvious prerequisites before suggesting routine genetic screening of rheumatic patients for IEIs. Additional parameters that would be relevant to the genetic screening for IEIs in rheumatic disorders include the availability and cost of genetic testing. With respect to the cost, it should be borne in mind that through a high throughput sequencing approach additional biomarkers of rheumatic disease, such as HLA-DR4 or HLA-B27 can be evaluated. An additional complexity of routine genetic testing would stem for the identification of variants of uncertain significance (VUS), whose functional validation may be an arduous task, necessitating specialized assays or even the development of variant expressing cellular or animal models.

Familial HLH is a life-threatening condition, which even if managed successfully has a high recurrence risk.^129^ Therefore, HSCT is the only curative treatment and diagnosis of a familial rather than a secondary form should lead to more aggressive treatment and timely consideration of HSCT. The clinical features of HLH in MAS can be identical to the ones of familial HLH.^123^ Besides genetic testing, markedly reduced or absent NK cell cytotoxicity suggests familial rather than secondary HLH. In case of patients with secondary HLH and reduced NK cell cytotoxicity, reversion of NK cell dysfunction during remission strongly suggests the secondary disease form. Reduced activation-induced NK cell and cytotoxic T cell degranulation, evaluated through the measurement of surface levels of the lysosomal protein CD107a, can lead to the diagnosis of a familial form of HLH, i.e. a degranulation defect.

### Possible clinical consequences from the identification of inborn errors of immunity among patients with rheumatic disorders

Identification of the genetic and consequently pathophysiological disease basis of IEI has led to targeted therapeutic approaches, especially in order to treat manifestations of immune dysregulation. In particular, diagnosis of CTLA-4 insufficiency or LRBA deficiency has led to treatment with CTLA-4-Ig (Abatacept®).^130^^,^^131^ STAT1 or STAT3 gain-of-function-associated immune dysregulation has been successfully treated with JAK inhibitors,^132^ whereas selective phosphoinositide-3-kinase δ (PI3Kδ) inhibitors represents an efficacious therapeutic option in patients with activated phosphoinositide 3-kinase δ syndrome (APDS).^133^ In case of autoinflammatory disorders, JAK inhibitors have been employed to control type I IFN-mediated responses and IL-1 inhibitors to restrict the consequences of the hyperactivation of the inflammasome.^134^ Also diagnosis of many of previously discussed novel IEIs has led or could lead to targeted treatment (Table 1). However, the broad biological consequences of some genetic defects, especially those affecting transcription factors (e.g. Helios) or epigenetic modification (e.g. TET2) are difficult to revert through a targeted treatment.

**Table 1:** Novel inborn errors of immunity (IEIs) causing immune dysregulation and possible targeted treatments.

Gene	Inheritance	Clinical features	Tried/hypothetical (marked with “?”) targeted treatment	Reference
ATAD3A deficiency	AD/AR	Systemic sclerosis-like disease, neurological defects (development delay, spasticity)	JAK inhibitors, inhibiting at least JAK1 (baricitinib/ruxolitinib) STING inhibitors (e.g. SN-011)? IFNAR1-blockade (e.g. anifrolumab)?	^135^^,^^136^
CEBPE gain-of-function/neofunction	AR	Recurrent abdominal pain, pyoderma gangrenosum, granulomas, aseptic fever, abscesses	IL-1/IL-18 inhibitors? Nonselective JAK inhibitors?	
ELF4 deficiency	XL	Systemic autoinflammation with *Behçet's-like* orals ulcers, SLE-like rash, IBD, aseptic fever	IL-1 inhibitors TNF inhibitors IL12p40 blockade	^28^
HCK gain-of-function	AD	Cutaneous vasculitis, eczema, interstitial lung disease	Broad inhibition of cytokine signaling through nonselective JAK inhibitors? HCK-specific inhibitor (e.g. RK20449)	^137^
Helios deficiency	AD/AR	Immunodeficiency (upper and lower respiratory infections, thrush), mucosal ulcers, lymphadenopathy, SLE, ITP, AIHA, EBV-associated HLH, lymphoma	HSCT (risk for HLH/lymphoma) (standard SLE treatment (HCQ, steroids) might be efficacious in some patients)	68, 69, 70
Ikaros gain-of-function	AD	Polyautoimmunity (diabetes, colitis, thyroiditis, autoimmune cytopenias), lymphoproliferation, features of IgG4-related disease (IgG4 positive plasma cell expansion)	HSCT? Autologous T cell gene therapy (such as performed for CTLA-4 insufficiency)?	^138^
LSM11 deficiency	AR	AGS	JAK inhibitors, inhibiting at least JAK1 (baricitinib/ruxolitinib) STING inhibitors (e.g. SN-011) IFNAR1-blockade (e.g. anifrolumab)?	^135^^,^^136^
NCKAP1L deficiency	AR	Immunodeficiency with recurrent upper respiratory tract infections, eczemas, cutaneous abscesses, atopy, ulcers, lymphadenopathy, anti-dsDNA antibodies, aseptic fever, features of HLH	HSCT (as in other actinopathies)? Small molecule activating Arp2/3?	^111^^,^^114^
NEMO exon 5 deletion	XL	Fever, skin rash, granulomas, granulomas, uveitis, sterile osteomyelitis CNS involvement, panniculitis, hepatosplenomegaly, ectodermal dysplasia	Nonselective JAK inhibitors?	^110^
PD-1 deficiency	AR	Polyautoimmunity, including endocrinopathies, JIA and severe ILD, tuberculosis	HSCT? Autologous T cell gene therapy (such as performed for CTLA-4 insufficiency)? Inhibition of TCR proximal signaling (Lck-inhibitors? ZAP-70 inhibitors?)	^138^
PSMB9 gain-of-function	AD	Neonatal-onset fever, eczemas, juvenile dermatomyositis, pulmonary hypertension, basal ganglia calcification and immunodeficiency	Nonselective JAK inhibitors HSCT with/without thymus transplantation? Selective inhibitors of the immunoproteasome (e.g. ONX-0914 or KZR-616)?	^139^
RIPK1 deficiency (AD)	AD	Recurrent aseptic fever, lymphoproliferation, ulcers	IL-6 blockade (e.g. tocilizumab) IL-1/IL-18 blockade?	^98^^,^^99^
RNU7-1 deficiency	AR	AGS	JAK inhibitors, inhibiting at least JAK1 (baricitinib/ruxolitinib) STING inhibitors (e.g. SN-011) IFNAR1-blockade (e.g. anifrolumab)?	^135^^,^^136^
SOCS1 haploinsufficiency	AD	Early-onset polyautoimmunity, ITP, AIHA, SLE, GN, psoriasis, arthritis, thyroiditis, hepatitis, hepatosplenomegaly, recurrent bacterial infections	Nonselective JAK inhibitors? HSCT (obstacle: non-hematopoietic expression of SOCS1)?	81, 82, 83
STAT2 R148 LOF/regulation	AR	Severe early-onset autoinflammation	JAK inhibitors, inhibiting at least JAK1 (baricitinib/ruxolitinib) interferon α receptor 1 (IFNAR1)-blockade (e.g. anifrolumab)?	^87^
STING-associated vasculopathy, infantile onset (SAVI)	AD/AR	Early onset fever, ILD, polyarthritis and cutaneous vasculitis	JAK inhibitors, inhibiting at least JAK1 (baricitinib/ruxolitinib) STING inhibitors (e.g. SN-011) IFNAR1-blockade (e.g. anifrolumab)?	^102^^,^^135^^,^^140^
SYK gain-of-function	AD	Arthritis, eczema, vasculitis, colitis, CNS inflammation, recurrent infections	HSCT (partial treatment of mice with Syk gain-of-function, likely due to the role of SYK in innate immune sensing by non-hematopoietic cells, such as intestinal epithelial cells) and/or R406 (SYK-specific inhibitor)	^33^
TBK1 deficiency (AR)	AR	CNS inflammation, arthritis and vasculitis	TNF inhibitors	^108^
TET2 deficiency	AR	*ALPS-like autoimmunity and lymphoproliferation*, EBV viremia, lymphoma	HSCT?	^91^^,^^92^
TLR8 gain-of-function	XL/somatic gain-of-function mutations in *TLR8*	Neutropenia, hypogammaglobulinemia, lymphoproliferation, autoimmunity and bone marrow failure	HSCT (esp. in case of prolonged or unresponsive neutropenia), CU-CPT9 or alternative human TLR8 inhibitor?	^141^
Vacuoles, E1 enzyme, X-linked, autoinflammatory, somatic (VEXAS) syndrome	somatic gain-of-function mutations in *UBA1*	Treatment-refractory inflammatory syndrome with aseptic fevers, dysplastic bone marrow, cutaneous and pulmonary inflammation, nephritis, chondritis and various types of vasculitis	Broad inhibition of cytokine signaling through nonselective JAK inhibitors? HSCT to remove progenitor cells harboring pathogenic UBA1 mutation with/without reversion of mechanism that accounts for somatic mutations in UBA1	^142^

AD, autosomal dominant; AGS, Aicardi-Goutières syndrome; AIHA, autoimmune hemolytic anemia; ALPS, autoimmune lymphoproliferative syndrome; AR, autosomal recessive; CNS, central nervous system; GN, glomerulonephritis; HCQ, hydroxychloroquine; HLH, hemophagocytic lymphohistiocytosis; HSCT, hematopoietic stem cell transplantation; IBD, inflammatory bowel disease; IFNAR1, interferon α receptor 1; IL, interleukin; ILD, interstitial lung disease; ITP, immune thrombocytopenic purpura; JAK, Janus kinase; JIA, juvenile idiopathic arthritis; SLE, systemic lupus erythematosus; TCR, T cell receptor; TNF, tumor necrosis factor; XL, X-linked.

Abatacept® and JAK inhibitors are already licensed treatments to treat rheumatic diseases.^143^^,^^144^ However, they are not first-line treatments and will be considered only after failure of conventional synthetic DMARDs (csDMARDs), which may precipitate the onset of hypogammaglobulinemia or immunodeficiency in patients with an underlying IEI. Therefore, on the occasion of rheumatic disease due to one of the aforementioned disorders, prioritizing an otherwise second-line targeted treatment should be considered. Given the phenotypic complexity of IEIs, especially of disorders associated with immune dysregulation, diagnosis of an IEI in patients with rheumatic disorders may affect initial diagnostic as well as follow-up procedures, which depending on identified IEI should take into consideration the risk for particular infections, malignancies and extraarticular manifestations. Another possible consequence would be the handling of the risk for infections, in case of disorders affecting cellular and/or humoral immunity, whose diagnosis should lead to extra caution before introducing immunomodulatory regimens with a relatively stronger immunosuppressive effect, such as glucocorticoids, timely consideration of prophylactic anti-infective medications or immunoglobulin replacement.

## Conclusions and perspectives

The monogenic disease etiology in IEIs provides a relatively simple model for the pathogenesis of immune dysregulation, highlighting the central etiopathogenic role of single genes. Consequently, the discovery of rare IEIs has provided clues to the pathomechanisms of more common, though genetically complex, autoimmune and autoinflammatory disorders. To this end, we have presented the mechanisms of autoimmunity and autoinflammation in newly discovered IEI, that may be relevant for the pathogenesis of rheumatic disorders.

Given the emerging recognition of the fact that IEI can manifest as a well-classified rheumatic disorder, distinguishing rheumatic patients with an underlying IEI, can become a therapeutically relevant challenge in clinical daily practice (Fig. 2). Besides clinical or laboratory evidence of immunodeficiency, such as hypogammaglobulinemia or disturbed B cell differentiation, unusual extraarticular manifestations in case of RA or a spondyloarthritis (SpA) and of course, family history, focusing on all likely manifestations of IEI (i.e. immunodeficiency, immune dysregulations and malignancies) may aid the identification of rheumatic patients with an underlying IEI through genetic testing, which however, needs to be further investigated.

### Outstanding questions

1. The monofactorial, i.e. single-gene concept of IEIs has been challenged by the variable expressivity and the incomplete penetrance of relatively common IEIs, especially of those falling under disorders of immune dysregulation. The latter suggests the pathogenic role of additional genetic, epigenetic, environmental and/or lifestyle-related factors in the pathogenesis of immune dysregulation, which have been barely studied. Identification of the factors affecting the phenotypic outcome of an otherwise disease-causing variant could provide additional insight to the pathogenesis of rheumatic disorders and indicate additional treatment strategies.
2. Evidence derived mainly from retrospective evaluation of series of treated patients suggests the efficacy of targeted treatments in controlling IEIs-associated immune dysregulation. However, controlled clinical trials on the safety and efficacy of such targeted therapies remain scarce. Further, in several IEIs, small molecule inhibitors that specifically target affected molecules or signaling pathways need to be developed.
3. A subset of patients diagnosed with rheumatic disorders harbor IEI-causing variants, whose identification could lead to the employment of a targeted treatment. The proposed red flags that indicate an underlying IEI are based on the characterization of diagnosed IEI-patients. Given the increasing number of IEIs and the variable expressivity of disorders of immune dysregulation, unbiased sequencing studies on patients with rheumatic disorders are needed to firmly establish the red flags of an underlying IEI and consequently evaluate the role of routine genetic testing in rheumatic disorders.
  **Search strategy and selection criteria**
  The search strategy of this review entails the most current research papers published in major scientific journals, focusing on inborn errors of immunity of the table IV and VII added in the 2022 IUIS classification (reference 3), that manifest with rheumatic disease. For individual genes we performed search in “PubMed”. Our literature selection criteria are based on research works published by well-known experts in the field of inborn errors of immunity. Our own relevant literature has been also discussed in this review.

## Contributors

Writing—original draft preparation, G.S.; writing and editing, T.W.; original figure preparation, G.S. All authors have read and approved the final version of the manuscript and agreed to it's submission.

## Data sharing statement

Not applicable.

## Declaration of interests

The authors declare no conflict of interest.
